# The impact of surveillance and other factors on detection of emergent and circulating vaccine derived polioviruses

**DOI:** 10.12688/gatesopenres.13272.1

**Published:** 2021-06-11

**Authors:** Megan Auzenbergs, Holly Fountain, Grace Macklin, Hil Lyons, Kathleen M O'Reilly

**Affiliations:** 1 Centre for Mathematical Modelling of Infectious Diseases, London School of Hygiene & Tropical Medicine, London, WC1E 7HT, UK; 2 Polio Eradication, World Health Organization, Geneva, Switzerland; 3 Institute for Disease Modeling, Bellevue, Washington, USA

**Keywords:** polio, vaccination, eradication, cVDPVs, OPV

## Abstract

**Background:** Circulating vaccine derived poliovirus (cVDPV) outbreaks remain a threat to polio eradication. To reduce cases of polio from cVDPV of serotype 2, the serotype 2 component of the vaccine has been removed from the global vaccine supply, but outbreaks of cVDPV2 have continued. The objective of this work is to understand the factors associated with later detection in order to improve detection of these unwanted events.

**Methods:** The number of nucleotide differences between each cVDPV outbreak and the oral polio vaccine (OPV) strain was used to approximate the time from emergence to detection. Only independent emergences were included in the analysis. Variables such as serotype, surveillance quality, and World Health Organization (WHO) region were tested in a negative binomial regression model to ascertain whether these variables were associated with higher nucleotide differences upon detection.

**Results:** In total, 74 outbreaks were analysed from 24 countries between 2004 and 2019. For serotype 1 (n=10), the median time from seeding until outbreak detection was 284 (95% uncertainty interval (UI) 284-2008) days, for serotype 2 (n=59), 276 (95% UI 172-765) days, and for serotype 3 (n=5), 472 (95% UI 392-603) days. Significant improvement in the time to detection was found with increasing surveillance of non-polio acute flaccid paralysis (AFP) and adequate stool collection.

**Conclusions:** cVDPVs remain a risk globally; all WHO regions have reported at least one VDPV outbreak since the first outbreak in 2001. Maintaining surveillance for poliomyelitis after local elimination is essential to quickly respond to both emergence of VDPVs and potential importations. Considerable variation in the time between emergence and detection of VDPVs were apparent, and other than surveillance quality and inclusion of environmental surveillance, the reasons for this remain unclear.

## Introduction

Polio has been targeted for eradication since 1988 when countries represented within the World Health Assembly committed to eradication ^1^. Whilst the initial goal to eradicate all poliovirus by 2000 was not achieved, two of the three wild serotypes have been eliminated, most recently type 3 in 2018 ^2– 4^. The main driver in this reduction of cases has been vaccination achieved through both routine and supplementary immunisation activities (SIAs), largely with the oral polio vaccine (OPV), a live attenuated vaccine. OPV is important for polio eradication, as it provides both humoral and intestinal immunity. However, the genetic instability of the attenuated virus can result in mutations that increase transmissibility and neurovirulence of infections ^5, 6^. Consequently, circulating vaccine-derived polioviruses (cVDPVs) can arise and cause paralysis in affected individuals. Prior to 2001, these outbreaks had not been reported in any countries using OPV ^7^, and recent analysis has suggested that cVDPV emergence and spread is more common in populations with low to moderate mucosal immunity against poliovirus ^8, 9^.

Since observing this unwanted effect of OPV vaccination, along with vaccine-associated paralytic polio (VAPP) and immunodeficiency-associated VDPVs (iVDPVs), removal of OPV from use has been prioritised within the Global Polio Eradication Initiative (GPEI) ^10, 11^. Especially for serotype 2, the risks of OPV have begun to outweigh the benefits because OPV use can seed additional outbreaks in susceptible populations, and the continued use of OPV2 was deemed unnecessary ^12^. The Switch from trivalent OPV (tOPV) to bivalent OPV (bOPV), removing serotype 2, was accomplished globally in a two-week period at the end of April 2016 ^13^. Instead of the anticipated decrease in circulating VDPVs, in the third- and fourth-years post-Switch, outbreaks and geographic spread of outbreaks have increased.

The strategy for eradication described in the 2013–2018 GPEI Strategic Plan outlines that wild poliovirus should be interrupted whilst strengthening immunization systems, including the introduction of inactivated polio vaccine (IPV) ^10^. Alongside, considerable investment has been made towards transition to a polio-free world that includes containment of all polioviruses, including minimising the risks of unintended release from laboratory facilities, and eventual removal of the OPV (known as cessation) ^14^. This transition phase is needed to ensure that the chances of poliovirus transmission in a susceptible population would be a low as manageable, and that populations would remain protected from outbreaks. The Polio Post-Certification Strategy ^14^, describes the many facets of containing polioviruses, protecting populations, cessation of the OPV and detecting and responding to a polio threat. The Switch from tOPV to bOPV provided the first trial of removing one of the serotypes from the global vaccine supply. Within the Polio Post-Certification Strategy, the pre-cessation (zero-to-one-year post-certification) and immediate post-cessation (two to five years post-certification) were regarded as the time periods where VDPVs were most likely to emerge, where the risk was thought to be highest 12–18 months after (in the most recent example) bOPV withdrawal. The period of time until detection is based on modelling which suggests that the cumulative probability of detecting circulating poliovirus is over 99.9% by four years ^15^, but the modelling did not account for weaknesses in surveillance or include specific aspects of VDPV transmission. 

cVDPVs are of particular concern in areas with low to moderate OPV induced immunity, as the virus is able to emerge and maintain transmission ^9, 16^. In (mostly high-income) countries with no OPV vaccination, there is minimal risk of VDPV emergence because the source is largely absent, transmission risk is lower, and vaccination coverage with the IPV is usually high. However, other risk factors for cVDPVs include: continued OPV use at low rates of coverage, prior elimination of the corresponding wild poliovirus serotype, insensitive acute flaccid paralysis (AFP) surveillance, and use of monovalent OPV (mOPV) and bOPV in SIAs due to the emergent risk of the live attenuated vaccine ^6, 8, 17^. A novel, genetically stable OPV2 that is a modified version of the existing OPV2 but better retains attenuation is currently in development and has been approved and deployed for emergency use in 2021 in order to mitigate these risk factors ^18, 19^.

Here we provide a retrospective analysis of cVDPV outbreaks between 2004 and 2019 and estimate the time from emergence to detection using publicly available data. We explore the differences in time to detection across VDPV serotypes and examine the effect of AFP surveillance and other factors on the time to detection. The aim is to provide useful information on the time to detection of VDPV outbreaks by serotype and the factors that affect this, in order to inform future cessation planning.

## Methods

Detection of poliomyelitis outbreaks are dependent upon global surveillance for AFP and the Global Polio Laboratory Network where clinical specimens are investigated to identify poliovirus as the causative agent. To confirm poliovirus infection, at least two stool specimens should be collected 24–48 hours apart and within 14 days of the onset of AFP in affected individuals ^20^. Sequencing of the VP1 region of the viral genome is used to classify poliovirus; if the sample differs from the parental OPV strain by 1–15% (or from 0.6% for serotype 2), the case is defined as a VDPV ^9, 21^. However, this definition changed in 2010 for serotype 2 only, such that prior to 2010, 10 nucleotide mutations in the VP1 region constituted a VDPV, but later on, the cut-off dropped to 6 nucleotide mutations. Therefore, we exclude type 2 outbreaks prior to 2010 (n=16) to account for this change as historic type 2 outbreaks where the isolate had <10 nucleotide mutations would not have been counted as a cVDPV.

By definition, cVDPV refers to VDPV isolates for which there is evidence of person-to-person transmission in the community and ‘genetically linked VDPVs’ are isolated from at least two individuals who do not live in the same household, or from one individual and ≥1 environmental surveillance (ES) sample reported through the comprehensive surveillance network ^11^. Within the GPEI surveillance network, cVDPV outbreaks that spread across country borders are treated as separate outbreaks (requiring a response within each country). Here we are only interested in the emergence of new cVDPV outbreaks, and exclude outbreaks as a result of international spread. For example, an emergence first detected in Jigawa State, Nigeria, which has spread to several countries in West Africa is only included once in the dataset. Where possible, the lineage code for each cVDPV2 emergence is provided (extended data Table 1 ^22^). 

Poliomyelitis is a notifiable disease, and as part of global surveillance for poliomyellitis, the GPEI and WHO laboratories report all confirmed outbreaks thorugh the Morbidity and Mortality Weekly Reports (MMWR). Consequently, we use these reports to compile a spreadsheet of all cVDPV outbreaks from 2000 to February 2020. Outbreaks were first identified using MMWR reports and then country and year(s) of the outbreak were searched using the search terms: ‘vaccine-derived poliovirus* OR VDPV OR circulating vaccine-derived poliovirus* OR cVDPV’. This search criteria is not a systemtic review of all literature for polio outbreaks within the time period, but due to the nature of disease surveillance for poliomyelitis, this resulted in a comprehensive list of outbreaks. The number of nucleotide sequences that are different to the Sabin 2 strain at first detection (referred to as ‘VP1 divergence’) and the dates of the first and last isolates of the outbreak were also collated through the literature search. As per exclusion criteria, we did not include outbreaks that did not meet the aforementioned cVDPV definition or were the result of international spread. The annual country-level non-polio acute flaccid paralysis (AFP) rate and percentage of adequate stool specimens collected, both indicators of surveillance quality, were extracted for each outbreak and year corresponding to the start of the outbreak. In order examine the effect of environmental sampling as a supplement to AFP surveillance, the mechanism via which the first isolate was detected (AFP or ES) was ascertained for each outbreak. Additionally, we included WHO region, Diphtheria-Pertussis-Tetanus vaccine dose 3 (DPT3) coverage (which is often used as a marker for routine immunisation coverage), and whether the outbreak was detected before/after 2016. Multiple independent emergences observed within the same country-year unit of observation were treated as multiple observations even if the associated surveillance data and outbreak response remained the same. HF was responsible for initial database creation while MA independently cross-checked the data.

Variables associated with the number of nucleotide differences were explored using a negative binomial model. A negative binomial model was selected because the variance of the reported number of nucleotide differences was larger than the mean and the data was highly dispersed. The minimum number of mutations was 9 for serotypes 1 and 3, and 6 for serotype 2, and the outcome variable was shifted-left so that the minimum number was 0. Separate datasets were created for serotype 2 and serotypes 1 and 3, and the latter retained a covariate for serotype. Preliminary analysis illustrated that outbreaks with nucleotide mutations ≥30 (n=4) affected the fit of the model to the data (due to overdispersion that could not fully be accounted for) and were removed from the dataset as outliers. A multivariate regression model was built using stepwise removal by comparing differences in the Akaike information criteria (AIC) between candidate models and assessing the negative binomial dispersion parameter (θ). Interactions between variables were also examined.

For every VDPV outbreak, we estimate the time to detection using the following methods. Each VDPV outbreak has included with it the number of VP1 mutations associated with the first case(s), and is used to estimate the time to detection. The first VP1 mutation of the Sabin strain is assumed to be instantaneous and each subsequent mutation follows an average rate of 1.14×10 ^-2 ^nucleotides per site per year ^23, 24^. The VP1 RNA gene consists of 906 nucleotides, so we would expect approximately 1 nucleotide change every 35 days under a constant clock model. We assume that the viral evolution rate is the same across all serotypes ^25, 26^. Each independent mutation was modelled using an exponential distribution and the sum of waiting times as an Erlang distribution, as done for a previous analysis of cVDPVs ^24^. By treating each VDPV detection as a random sample of the population parameter for the time to detection, we use bootstrapping of the sample estimates of time to detection to provide robust estimates for serotype 2 and serotypes 1 and 3. The empirical distribution function of the bootstrapped samples were used to calculate the probability of VDPV outbreaks being detected within one and four years. All analyses were carried out in R version 4.0.3.

This work was completed between June 2020 and April 2021. This project received ethical approval from the London School of Hygiene & Tropical Medicine (LSHTM) on 29 ^th^ June 2020: project ID 21929.

## Results

### Independent cVDPV outbreaks

Review of MMWR reports identified a total of 96 outbreaks in 28 countries. However, once outliers were excluded and the change of cVDPV2 definition was accounted for and cVDPV2 outbreaks pre-2010 were removed, a total of 74 cVDPV outbreaks as a result of independent emergences were analysed from 24 countries ( Table 1). cVDPV type 2 was the most frequent serotype isolated, accounting for 80% of outbreaks, followed by serotypes 1 and 3, accounting for 13% and 7% of outbreaks, respectively. Of the 74 outbreaks, 18 (24%) were first detected via ES.

**Table 1.** Summary of all circulating vaccine derived polioviruses (cVDPVs) included in the analysis split by serotype. WHO=World Health Organization; NPAFP= non-polio acute flaccid paralysis; CI=confidence interval.

WHO Region	Country	Number of outbreaks	Median * duration days (range **)	Median * nucleotide difference from Sabin strain of the first isolate (range **)	Mean NPAFP rate (per 100,000 children <15) (95% CI)	Mean % adequate stool samples (95% CI)
**Serotype 1**						
AFR	MADAGASCAR	1	338	20	4.2	85.6
AFR	MOZAMBIQUE	1	112	27	2.7	87.2
AMR	DOMINICAN REPUBLIC	1	190	17	-	-
EUR	UKRAINE	1	7	20	2.7	97.4
SEAR	INDONESIA	1	139	10	2.4	85.5
SEAR	MYANMAR	2	258 (59, 458)	19.5 (14, 25)	2.8	92.7
WPR	CHINA	2	55 (51, 59)	11 (9, 13)	1.9	92.2
WPR	LAOS	1	269	21	2.6	57.1
WPR	PAPUA NEW GUINEA	1	193	14	7.9	44.7
Global Type 1 total		10	125.5 (7, 458)	17 (9, 27)	3.2 (1.9, 4.5)	82.7 (70.2, 95.2)
**Serotype 2**						
AFR	ANGOLA	5	195 (39, 288)	7 (6, 10)	5.0	85.1
AFR	CENTRAL AFRICAN REPUBLIC	7	99 (0, 275)	7 (6, 10)	9.2	71.1
AFR	CHAD	2	184 (97, 270)	6 (6)	10.2	84.6
AFR	DRC	12	173 (1, 473)	8 (6, 19)	7.7	84.2
AFR	ETHIOPIA	4	94 (41, 151)	12 (10, 18)	2.9	90.8
AFR	GUINEA	1	475	12	2.6	96.6
AFR	MOZAMBIQUE	1	57	6	3.4	88.5
AFR	NIGERIA	9	84 (0, 637)	10 (6, 16)	11.9	95.4
AFR	SOUTH SUDAN	1	3	9	4.2	94.4
AFR	TOGO	1	78	13	4.6	70.2
AFR	ZAMBIA	1	71	9	3.8	84.3
EMR	AFGHANISTAN	1	1295	8	11.0	92.6
EMR	PAKISTAN	9	58 (8, 654)	6 (6, 9)	17.7	87.1
EMR	SYRIA	1	202	22	3.6	80.4
EMR	YEMEN	1	179	6	3.4	91.5
SEAR	MYANMAR	1	172	13	2.5	93.2
WPR	CHINA	2	300 (113, 487)	10 (6, 13)	2.0	92.3
Global Type 2 total		59	105 (0, 1295)	8 (6, 22)	8.9 (7.3, 10.4)	86.2 (84.2, 88.2)
**Serotype 3**						
AFR	ETHIOPIA	1	556	12	2.6	79.0
AFR	MADAGASCAR	1	32	13	1.3	89.4
EMR	SOMALIA	1	183	14	4.8	97.7
EMR	YEMEN	1	454	18	4.3	93.2
WPR	CAMBODIA	1	50	17	2.0	95.8
Global Type 3 total		5	255	14 (12, 18)	3.0 (1.2, 4.8)	91.0 (81.3, 100)
Total Outbreaks		74	109 (0, 1295)	9 (6, 27)	7.7 (6.3, 9.0)	86.0 (83.8, 88.2)

*Median and **range provided if more than 1 outbreak, otherwise single value provided

For serotype 1 (n=10), the median nucleotide divergence for the first isolate of the outbreak was 17 (range: 9, 27) and the mean non-polio AFP rate was 3.2 (95% confidence interval (CI): 1.9-4.5) ( Table 1). Half (50%) of type 1 outbreaks were contained or closed within 120 days. For serotype 2 (n=59), the median nucleotide divergence for the first isolate of the outbreak was 8 (range: 6, 22) and the mean non-polio AFP rate was 8.9 (95% CI: 7.3, 10.4). The majority of type 2 outbreaks (54%) were contained within 120 days. For serotype 3 (n=5), the median nucleotide divergence for the first isolate of the outbreak was 14 (range: 12, 18) and the mean non-polio AFP rate was 3.0 (95% CI: 1.2, 4.8) cases per 100,000 of the population under 15 years of age. In total, 40% of type 3 outbreaks were contained within 120 days.

For serotype 2, a regression model of the number of nucleotide differences of the first isolate for each outbreak suggests a decrease in nucleotide difference with increasing non-polio AFP rate and percentage of adequate stool samples collected (incidence rate ratio (IRR) 0.18, 95% CI 0.06-0.49, *p*<0.01 and IRR 0.91 95% CI 0.84-0.99, *p*=0.05, respectively), but no significant difference between classification (AFP or ES) ( *p*=0.07), Table 2. Interaction between non-polio AFP rate and percentage of adequate stool samples was significant for both serotype 2 and serotypes 1 and 3 (IRR 1.02 95% CI 1.01-1.04, *p*<0.01 and IRR 1.01 95% CI 1.0-1.03, *p*=0.03, respectively). A regression model was attempted for the 15 outbreaks that were either type 1 or 3, but low sample size prevents meaningful interpretation (extended data Table 2 ^22^). The mean estimates of the regression terms for serotypes 1 and 3 were similar in value to serotype 2 estimates, for example, there was no significant difference in surveillance classification for serotypes 1 and 3 (IRR 0.22, 95% CI 0.04-1.28, *p*=0.08), but the confidence intervals of the regression estimates were wide, likely due to low sample size.

**Table 2.** Final regression model of factors associated with the number of nucleotide differences of the first isolate of vaccine derived poliovirus (VDPV) outbreaks. Sample size and dispersion parameter (θ) for the serotype 2 model are reported. AFP=acute flaccid paralysis; ES=environmental surveillance; IRR=incidence rate ratio; CI=confidence interval.

Serotype 2 (n = 59) θ = 0.99			
**Variable**	**Factor**	**IRR, multivariable** **(95% CI)**	**P-value**
**Intercept**	-	-	
**Type of surveillance via which first isolate was detected (AFP** **case or ES)** AFP: n = 42 (71.1%) ES: n = 17 (28.8%)	ES (vs. AFP)	2.15 (0.93, 5.4)	0.069
**Unit increase of non-polio AFP rate (cases per 100,000** **children aged <15 years old)** Mean (95% CI): 8.9 (7.3, 10.4)	Linear term	0.18 (0.06, 0.49)	<0.01
**Percent of stool samples adequately collected** Mean (95% CI): 86.2 (84.2, 88.2) <80%: n = 9 (15%)	Linear term	0.91 (0.84, 0.99)	0.047
Unit increase of non-polio AFP rate * Percent of stool samples **adequately collected**	Interaction term	1.02 (1.01, 1.04)	<0.01

**Figure 1.**
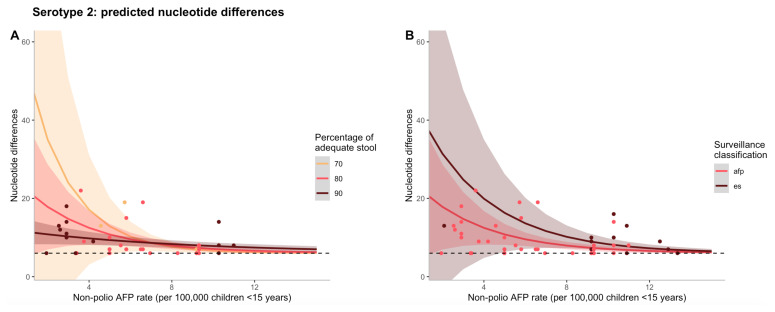
( **A**) Predicted counts of nucleotide differences for serotype 2 based on the final negative binomial regression model vs. non-polio AFP rate (per 100,00 children <15 years of age). The different colour lines correspond to varying percentages of adequate stool samples collected and the shaded regions represent a 95% confidence interval of model predictions. The different colour points also correspond to varying percentages of adequate stool samples collected, but represent data from a particular cVDPV2 outbreak. ( **B**) Predicted counts of nucleotide differences for serotype 2 based on the final negative binomial regression model vs. non-polio AFP rate (per 100,00 children <15 years of age). The different colour lines correspond to the type of surveillance via which the first isolate was detected and the shaded regions represent a 95% confidence interval of model predictions. The different colour points also correspond to the type of surveillance via which the first isolate was detected, but represent data from a particular cVDPV2 outbreak. In both figures, the black dashed line represents the minimum threshold cut-off of nucleotide differences (n=6) to be considered a cVDPV2.

The effects of non-polio AFP rate on nucleotide differences are shown in Figure 1, where the negative binomial regression model for serotype 2 is used to predict counts of nucleotide differences. To illustrate the interaction between non-polio AFP rate and percentage of adequate stool samples, Figure 1a illustrates that as both non-polio AFP rate and percentage of adequate stool increases, predicted nucleotide differences decline. Although the type of surveillance via which the first isolate was detected (AFP case or ES) was not significant in the final model and could act as a confounder, in Figure 1b, predicted nucleotide differences decrease as non-polio AFP rate increases for both surveillance mechanisms, but to a greater extent for AFP at low rates of non-polio AFP surveillance. 

**Figure 2.**
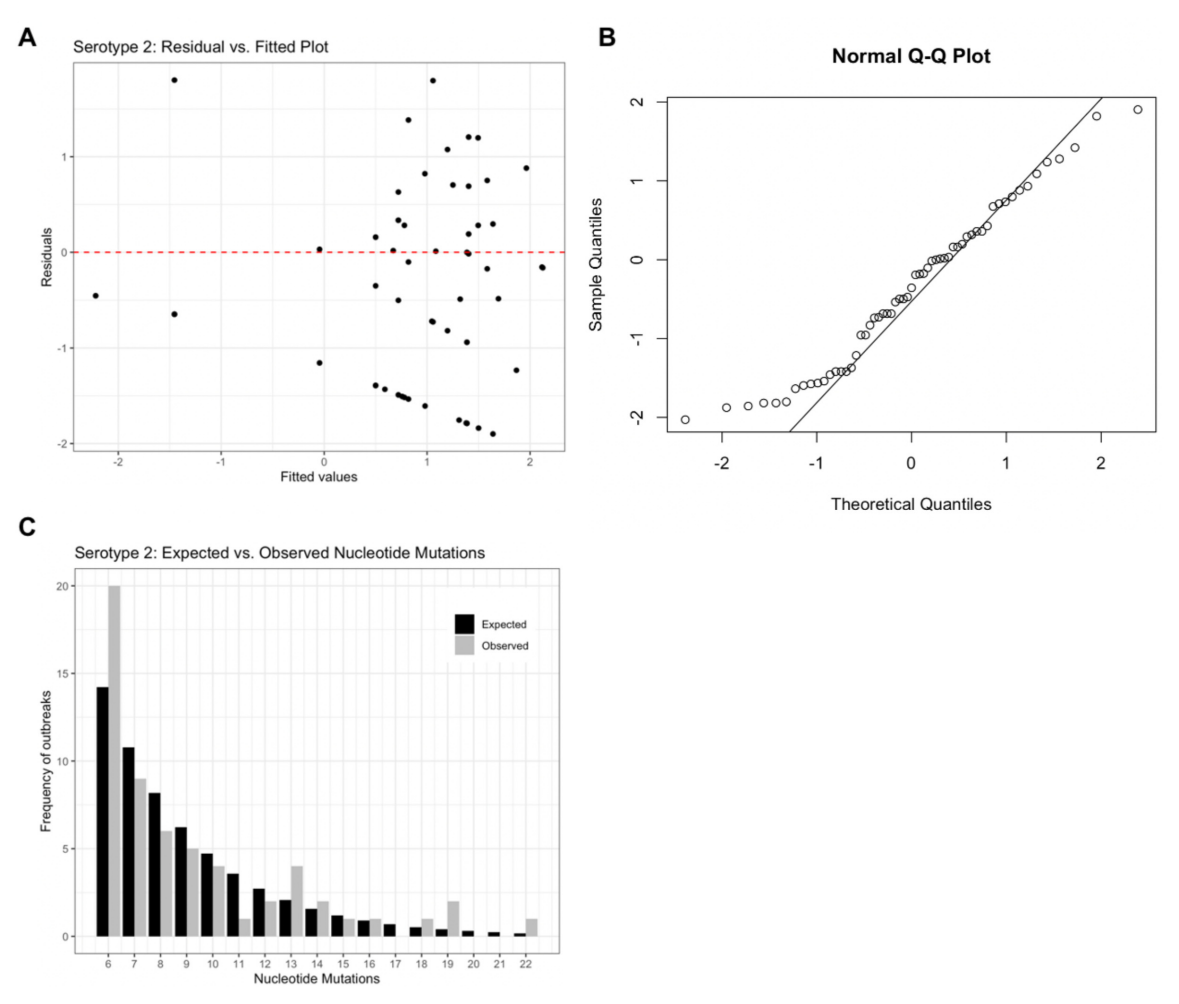
Serotype 2 diagnostic plots. ( **A**) residual vs. fitted values, ( **B**) Normal Q-Q plot and ( **C**) Expected vs. observed frequencies of nucleotide mutations assuming a negative binomial distribution.

Model residuals ( Figure 2a) for the serotype 2 model support an appropriate model structure as the plot illustrates homoscedasticity of the residuals. The Q-Q plot ( Figure 2b) further supports the assumed theoretical distribution for the final models as most values are centred along the Q-Q line, but the extreme values illustrate deviation from the assumed normal distribution of residuals. Figure 2c provides a visual comparison of expected vs. observed frequencies of nucleotide mutations. For serotype 2, outbreak frequencies corresponding to 6, 13, 19 and 22 nucleotide mutations are under-estimated by the model. Similar figures for serotypes 1 and 3 can be found in extended data Figure 1 ^22^.

### Estimating the time to outbreak detection

**Figure 3.**
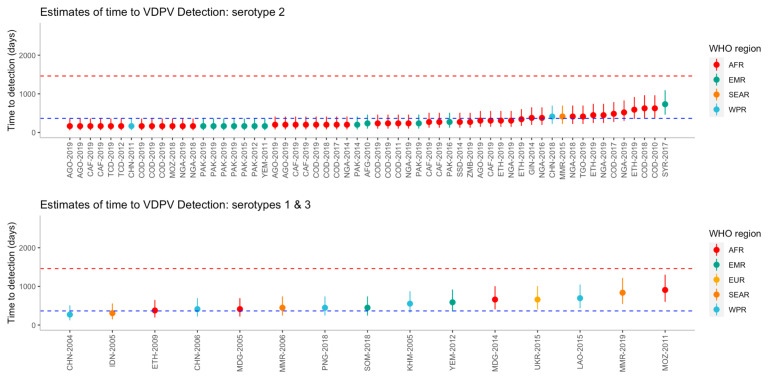
Estimated time to detection of each outbreak from the reported number of nucleotide differences from the Sabin strain, by serotype and region. Outbreaks are ordered on the x-axis by increasing time to detection, where uncertainty in the estimates are shown using 95% uncertainty intervals. Dashed lines represent one year (blue) and four years (red). Country names along the x-axis have been abbreviated using a country’s corresponding United Nations ISO 3166-1 alpha-3 code and year of first detection.

The time to detection was estimated for each outbreak, including uncertainty intervals ( Figure 3). Using the bootstrap method, the median time from seeding until outbreak detection for serotype 1 (n=10), was 284.3 (95% UI 284.3-2007.8) days and it was estimated that 91.5% of outbreaks would be detected within four years. The median time from seeding until outbreak detection for serotype 2 (n=59) was 276.1 (95% UI 172.3-764.8) days and 99.7% of outbreaks are estimated to be detected within four years. For serotype 3 (n=5), the median time from seeding until outbreak detection was 472.4 (95% UI 392.1-603.1) days and it was estimated that 100% of outbreaks would be detected within four years. Using the full uncertainty of the estimated time to detection, 20 of the 59 (34%) outbreaks of serotype 2 were detected under one year, whereas no serotype 1 or 3 outbreaks were detected within one year.

## Discussion

Polio eradication has been deemed an achievable undertaking, but with timeline and budget pressures ever present, it is importance to better understand the risks associated with cessation strategies and how to better plan for unwanted events. Emerging and circulating VDPVs are one of many threats to eradication, and detecting cVDPVs early in order to respond and limit transmission in communities will be important throughout the final stages of eradication.

This analysis illustrates several observations about cVDPV outbreaks. cVDPVs caused by serotype 2 have been more commonly detected than outbreaks caused by serotypes 1 and 3. This observation was apparent between 2000–2015 when the trivalent OPV was in use, as well as in subsequent years. When children are vaccinated with the OPV, the serotype 2 strain is more competitive in the gut mucosa ^27, 28^, resulting in increased ‘take’ by vaccinated individuals and subsequently a higher rate of secondary spread. The increased rate of spread was exacerbated post-Switch as a larger proportion of populations were not vaccinated with the serotype 2 strain due to the strategy of cessation. Additionally, a recent modelling study using inference from data on several clinical trials suggests that the order of transmissibility within equivalent populations is in the descending order of serotype 2, 1, and 3, which would further explain the observed frequency of each serotype-specific VDPV outbreak ^29^.

The number of nucleotide differences at the time of detection did not significantly vary between serotypes. For serotype 1, it has between estimated that there are approximately 200 infections for every case, 2000 infections for a serotype 2 case, and 1000 infections for a serotype 3 case ^30^. Based on differences in the asymptomatic rate, one might expect nucleotide differences of type 1 and 3 to be lower than serotype 2 when first detected, which was not observed. Based on the data from reported outbreaks, detection of cVDPVs does not seem sensitive to differences in symptomatic reporting that is associated with serotype, but may be influenced by unknown differences in where serotype specific detections emerge, which in-turn are affected by surveillance efforts within these countries.

The relationship between non-polio AFP rate and time to detection illustrates that in order to detect VDPVs early, a country needs to maintain a high rate of non-polio AFP surveillance. Now that wild poliovirus has been eliminated from the African continent, there may be incentive to reduce the intensity of non-polio AFP surveillance in the region. However, in line with the Global Polio Eradication Initiative Strategic Plan 2019–2023, which calls for closing gaps and strengthening global surveillance, this analysis has illustrated the importance of maintaining a high rate of non-polio AFP surveillance, especially for timely detection of cVDPVs ^11^. Therefore, as cVDPVs remain a threat, AFP surveillance must remain high in all areas with OPV use and/or suboptimal IPV coverage.

Adequate stool describes both the timeliness and quality of the samples (i.e., collected within 14 days of paralysis onset, 24–48 hours apart, and arrival at the laboratory in “good” condition) and current WHO guidelines state that at least 80% of AFP cases should have stool collection described as adequate, which this analysis further supports ^31^. However, while the mean percentage of adequate stool specimens in this analysis exceeds 80% for all serotypes, 15% and 20% of outbreaks of serotypes 2 and serotypes 1 and 3, respectively, fall below this targeted 80%. Also, this indicator is often reported at the national level while research suggests that percentage of adequate stool specimens is not only disparate at subnational levels, but particular age groups are not well-covered by the surveillance system and some countries report inaccurate rates of adequate stool specimen collection ^32^. After accounting for factors other than WHO regions, WHO region did not remain a significant explanatory variable, suggesting region specific differences do not account for nucleotide divergences as much as surveillance quality (both non-polio AFP rate and percentage of adequate stool samples collected).

In countries where ES is present, detection of emergent cVDPVs has previously been shown to be quicker than if surveillance relied on AFP alone ^33^. Although WHO region did not account for differences in detection time, ES is more commonly implemented across the AFR and EMR regions in comparison to other WHO regions. The total number of active ES sites across AFR, EMR and SEAR WHO regions was 620 in 2020, a 15% increase in the number of reported active ES sites in 2019 ^34^, but the percentage of the population within a catchment area remains comparatively low and poorly measured. While ES remains a useful source for detecting circulating viruses, its low coverage will mean that ES can only supplement AFP surveillance to enable rapid detection of VDPVs.

Of the cVDPV2 outbreaks that were seeded post-Switch, the source of about 95% of isolates was found to be consistent with mOPV2 outbreak response campaigns ^24^. This has been due to the inherent nature of mOPV2, and likely poorly implemented campaigns, and also because children recently vaccinated with mOPV2, or their contacts, travelled outside the response zones to areas where children born after the Switch were fully susceptible to infection ^35^. The need to improve these response campaigns has been recognised with an addendum to the Polio Endgame Strategy 2019–2023, whereby the strategy is to implement actions such as enhanced outbreak response campaigns and ensure sufficient supply of mOPV2 to diminish immunisation gaps ^10^. The novel OPV2 vaccine is expected to replace the mOPV in 2021–2022 (subject to findings during emergency use licensure), reducing the risk of cVDPV2 emergence. As illustrated in this analysis, emergences of cVDPV2 from mOPV2 are likely to continue for up to four years after the last mOPV2 campaign, meaning that nOPV2 use in outbreak response will be required for at least this period of time.

A weakness of our approach is that we assume that VDPV mutations occur at a constant and independent rate. In reality, multiple mutations may result in a reduction in nucleotide divergence (through back mutations). Consequently, our estimates may under-estimate the time to detection. Additionally, we have not used data on ambiguous (aVDPVs – progenitors to cVDPVs) to observe the frequency of detection across WHO regions. Inclusion of this data may provide further insight on factors associated with detection, but is reliant on consistent laboratory reporting of aVDPVs across WHO regions. Additionally, this analysis was a retrospective analysis of cVDPV outbreaks where few countries have included IPV into routine immunisation, meaning that we were unable to explore any effects of IPV on VDPV detection.

In conclusion, this analysis of cVDPV outbreaks illustrates that surveillance for AFP—ensuring a high non-polio AFP rate with adequate stool collection—can result in quick detection of cVDPV outbreaks, having the potential to prevent transmission and subsequent cases in populations. In all regions, undetected circulation of poliovirus will remain an issue until the current OPV vaccines are no longer necessary.

## Data availability

### Underlying data

Zenodo: mauzenbergs/polio_vdpv: VDPV Extended Data. https://doi.org/10.5281/zenodo.4776357 ^22^.

This project contains the following underlying data:

- VDPV_dat.csv

### Extended data

Zenodo: mauzenbergs/polio_vdpv: VDPV Extended Data. https://doi.org/10.5281/zenodo.4776357 ^22^.

This project contains the following underlying data:

- VDPV_Extended_Data.pdf

Data are available under the terms of the Creative Commons Zero "No rights reserved" data waiver (CC0 1.0 Public domain dedication).

## Software availability

Source code available from: https://github.com/mauzenbergs/polio_vdpv

Archived source code at time of publication:

https://doi.org/10.5281/zenodo.4776357 ^22^

License: Creative Commons Zero "No rights reserved" data waiver (CC0 1.0 Public domain dedication).
