# Supplementary material for: The impact of surveillance and other factors on detection of emergent and circulating vaccine derived polioviruses
Source: Gates Open Res. 2023 Apr 11;5:94. Originally published 2021 Jun 11. [Version 3] doi: 10.12688/gatesopenres.13272.3 (PMC8913522; doi:10.12688/gatesopenres.13272.3)
Supplement: Peer review history

# Peer review: Reviewer response for version 3

**DOI:** 10.21956/gatesopenres.15943.r33020

**Published:** 2023-04-17

**Authors:** Walter A. Orenstein

**Affiliations:** 1 Department of Epidemiology, Rollins School of Public Health, Emory University, Atlanta, GA, USA

The authors have addressed well the points made in this reviewer's intial review. There are a couple of issues not addressed that would make the manuscript more understandable to a "non-modeling" audience. The authors mention in their comments for Table 2 that a footnote was added to explan what IRR means. This reviewer did not see the footnote in the text.

 Second, on page 7, top of the right most column, there is a typo. "Importance" should be "important".

 For the legend of Figure 2, it would be helpful to have a definition of what a residual value is versus a fitted value.

 Otherwise, this is a very important manuscript and should be indexed, once the points above are addressed.

Is the work clearly and accurately presented and does it cite the current literature?

Yes

If applicable, is the statistical analysis and its interpretation appropriate?

I cannot comment. A qualified statistician is required.

Are all the source data underlying the results available to ensure full reproducibility?

Yes

Is the study design appropriate and is the work technically sound?

Yes

Are the conclusions drawn adequately supported by the results?

Yes

Are sufficient details of methods and analysis provided to allow replication by others?

Partly

Reviewer Expertise:

Polio Epidemiology and Polio Vaccination Policy

I confirm that I have read this submission and believe that I have an appropriate level of expertise to confirm that it is of an acceptable scientific standard.

# Peer review: Reviewer response for version 2

**DOI:** 10.21956/gatesopenres.14920.r32023

**Published:** 2022-06-07

**Authors:** Svea Closser

**Affiliations:** 1 Department of International Health, Johns Hopkins Bloomberg School of Public Health, Baltimore, MD, USA

I appreciate the authors' careful consideration of the reviewers' comments.

Is the work clearly and accurately presented and does it cite the current literature?

Yes

If applicable, is the statistical analysis and its interpretation appropriate?

I cannot comment. A qualified statistician is required.

Are all the source data underlying the results available to ensure full reproducibility?

Yes

Is the study design appropriate and is the work technically sound?

Yes

Are the conclusions drawn adequately supported by the results?

Yes

Are sufficient details of methods and analysis provided to allow replication by others?

Yes

Reviewer Expertise:

I am an anthropologist who has been studying the polio eradication program for more than 15 years.

I confirm that I have read this submission and believe that I have an appropriate level of expertise to confirm that it is of an acceptable scientific standard.

# Peer review: Reviewer response for version 1

**DOI:** 10.21956/gatesopenres.14511.r31648

**Published:** 2022-03-10

**Authors:** Svea Closser

**Affiliations:** 1 Department of International Health, Johns Hopkins Bloomberg School of Public Health, Baltimore, MD, USA

This is a very clearly written and well presented paper about the critically important issue of cVDPV outbreaks, with the conclusions presented logically and reasonably. Although I am not an expert in quantitative methods, the methods used appear logical and appropriate, and the authors understand well the nature of the dataset they are working with. The information and analysis presented are informative and practically useful. The discussion is a clear and helpful review of the available information on cVDPV outbreaks, and articulates well what this paper adds.

 A few suggestions for further strengthening an excellent and informative paper:

 Some of the main practical take-aways (that ES may be less immediately useful than high quality AFP surveillance; that lower quality AFP surveillance means that outbreaks are likely to continue undetected for a significant period of time; and that mop up campaigns using mOPV2 carry risks of seeding cVDPV outbreaks) could be highlighted more in the abstract.

 The implications could be pulled out a bit more in the Discussion—there could be some more courageous discussion here. Some questions that came up for me included: Given that surveillance across much of the world is not good enough to detect outbreaks on the timescale we all would like, what level of undetected transmission might we need to assume is ongoing globally, and what are the implications of this? What strategies might the authors suggest instead of mOPV2 mop ups? What is known about the extent to which nOPV, or IPV, would alleviate the cVDPV problem, and what might this paper add to discussions about prioritizing one vaccine over another?

Is the work clearly and accurately presented and does it cite the current literature?

Yes

If applicable, is the statistical analysis and its interpretation appropriate?

I cannot comment. A qualified statistician is required.

Are all the source data underlying the results available to ensure full reproducibility?

Yes

Is the study design appropriate and is the work technically sound?

Yes

Are the conclusions drawn adequately supported by the results?

Yes

Are sufficient details of methods and analysis provided to allow replication by others?

Yes

Reviewer Expertise:

I am an anthropologist who has been studying the polio eradication program for more than 15 years.

I confirm that I have read this submission and believe that I have an appropriate level of expertise to confirm that it is of an acceptable scientific standard.

# Response

**Published:** 2022-04-26

**Authors:** Megan Auzenbergs

**Affiliations:** London School of Hygiene & Tropical Medicine, UK

Thank you for your favourable critique of this manuscript. We have taken your comments into consideration and offer the following replies and changes: 

*1.    Some of the main practical take-aways (that ES may be less immediately useful than high quality AFP surveillance; that lower quality AFP surveillance means that outbreaks are likely to continue undetected for a significant period of time; and that mop up campaigns using mOPV2 carry risks of seeding cVDPV outbreaks) could be highlighted more in the abstract.*

- These are very helpful take-away points. Several changes have been made to strengthen messaging in the abstract, lines 28-31. As word count is limited for the abstract, we have tried to incorporate your feedback in the most concise, yet meaningful, way possible. 

*2.    The implications could be pulled out a bit more in the Discussion—there could be some more courageous discussion here. Some questions that came up for me included: Given that surveillance across much of the world is not good enough to detect outbreaks on the timescale we all would like, what level of undetected transmission might we need to assume is ongoing globally, and what are the implications of this? What strategies might the authors suggest instead of mOPV2 mop ups? What is known about the extent to which nOPV, or IPV, would alleviate the cVDPV problem, and what might this paper add to discussions about prioritizing one vaccine over another?*

- Several of your points have now been incorporated into the discussion section, specifically, your points around persistent low rates of AFP surveillance and ongoing undetected transmission:
  - "Low rates of non-polio AFP surveillance that persist across many settings coupled with the low case to infection rate for polio means undetected transmission is possible in many areas, jeopardising the attainment of polio eradication. As the risk of importation of infection across the African continent increases following the 2021 WPV1 importation in Malawi, adopting strategies to improve surveillance are increasingly important.”

# Peer review: Reviewer response for version 1

**DOI:** 10.21956/gatesopenres.14511.r30931

**Published:** 2021-08-06

**Authors:** Walter A. Orenstein

**Affiliations:** 1 Department of Epidemiology, Rollins School of Public Health, Emory University, Atlanta, GA, USA

This is an important study which provides modeling data showing earlier detection of cVDPV polio outbreaks at sites with better non-polio acute flaccid paralysis (NPAFP) reporting and adequate stool collections. This information can play a critical role in assuring if cVDPV outbreaks were occurring, they would be detected quickly, making containment easier.  

 The article could benefit with some discussion of why higher NPAFP rates well beyond the minimal threshold for quality are more predictive of earlier cVDPV detection. Does this mean that the standard is too low and are there data the authors can reference regarding what would represent accurate rates of a clinical syndrome not associated with polioviruses? Also, for Table 1, can the authors clarify what the time frame is for the NPAFP rate compared to 1st detection of the outbreak?

 Table 2 is important in showing the relationship between various factors and detection of cVDPVs at an earlier time. The IRR is unclear. It would be helpful to add a footnote to the table describing what an IRR of 2.15 in Environmental versus versus AFP surveillance means.

 The relationship of AFP versus ES for early detection is, as noted by the authors, not significant (p value 0.069). But this is really borderline non-significant and this reviewer feels the authors need to be cautious in dismissing the relationship.

 Figure 2 A and B are difficult for this non-statistician to understand. It would be helpful if more description of the graphs for a non-statistical audience was added.

 In the abstract, the authors mention the first outbreak in 2001 of cVDPV1. In actuality, the outbreak started in 2000 and continued into 2001. See MMWR 50(39) 855-6, October 5, 2001.

 On page 3, left column, 3rd paragraph, line 11, there is a typo: "a low" should be "as low".

 There is another typo on page 4, left column, line 10. The authors say "systemtic". Should it be "systematic"?

 Also in that same paragraph, there are some asterisks. This reviewer did not see what the asterisks referred to.

Is the work clearly and accurately presented and does it cite the current literature?

Yes

If applicable, is the statistical analysis and its interpretation appropriate?

I cannot comment. A qualified statistician is required.

Are all the source data underlying the results available to ensure full reproducibility?

Yes

Is the study design appropriate and is the work technically sound?

Yes

Are the conclusions drawn adequately supported by the results?

Yes

Are sufficient details of methods and analysis provided to allow replication by others?

Partly

Reviewer Expertise:

I have a career as a public health physician. I was Director of the US Immunization Program at the CDC for 16 years. For 3 years I was in charge of the polio efforts at the Bill & Melinda Gates Foundation (2008-2011)

I confirm that I have read this submission and believe that I have an appropriate level of expertise to confirm that it is of an acceptable scientific standard.

# Response

**Published:** 2022-04-26

**Authors:** Megan Auzenbergs

**Affiliations:** London School of Hygiene & Tropical Medicine, UK

We thank the reviewer for their insightful and positive critique. We have taken your comments into consideration and offer the following responses and changes to the manuscript:

*1.    The article could benefit with some discussion of why higher NPAFP rates well beyond the minimal threshold for quality are more predictive of earlier cVDPV detection. Does this mean that the standard is too low and are there data the authors can reference regarding what would represent accurate rates of a clinical syndrome not associated with polioviruses?* 

- Thank you for this useful question, this is a great point regarding the standards in place for AFP surveillance. As Tangermann et al. 2017 states: “high rates of AFP reporting do not necessarily imply highly sensitive surveillance, because, in the absence of sufficient supervision, there may be considerable over-reporting of children as AFP cases who actually do not have AFP, while true AFP cases.”
  - This suggests that while standards are in place, they perhaps do not accurately capture localised issues that may mitigate surveillance sensitivity. Accurate rates of clinical syndrome that are not associated with poliovirus (i.e., Guillain-Barré syndrome) would need to be detected with greater sensitivity to ensure true poliovirus cases are not missed. A change has been made to add this important point to the discussion, lines 345-351.
- Additionally, the most recent GPEI protocol for responding to poliovirus outbreaks describes NPAFP goals and how recommended levels of surveillance may vary across high-risk areas versus smaller areas with fewer children under 15 years of age. This is now included in the manuscript lines 355-360.

*2.    Also, for Table 1, can the authors clarify what the time frame is for the NPAFP rate compared to 1st detection of the outbreak?*

- The mean NPAFP rate that is listed in Table 1 is the annual rate reported during the year of the first detection of the outbreak. This information is provided in line 136:
  - “The annual country-level non-polio acute flaccid paralysis (AFP) rate and percentage of adequate stool specimens collected, both indicators of surveillance quality, were extracted for each outbreak and year corresponding to the start of the outbreak”.

*3.    Table 2 is important in showing the relationship between various factors and detection of cVDPVs at an earlier time. The IRR is unclear. It would be helpful to add a footnote to the table describing what an IRR of 2.15 in Environmental versus AFP surveillance means.*

- In the amended version, a footnote has been added to table 2 further explaining the interpretation this IRR and how the IRR can be used to predict what the nucleotide changes would be of an isolated virus under specific circumstances (AFP vs. ES).

*4.    The relationship of AFP versus ES for early detection is, as noted by the authors, not significant (p value 0.069). But this is really borderline non-significant and this reviewer feels the authors need to be cautious in dismissing the relationship.*

- Thank you for this important point. We agree that this relationship may have been incorrectly dismissed and we now make a point to mention the IRR despite the borderline non-significant p-value. Note that we have retained the finding in the model presented for this reason, but also that the IRR is positive, which points towards ES being implemented in high-risk areas with concerns for AFP rates. 
- We made a change in the manuscript to address your point, lines: 254-257 (or paragraph 3 of the results section).

*5.    Figure 2 A and B are difficult for this non-statistician to understand. It would be helpful if more description of the graphs for a non-statistical audience was added.*

- Thank you for this consideration, the caption for figure 2 A and B has now been updated with more explanation of how to best interpret the plots for a non-statistical audience.

*6.    In the abstract, the authors mention the first outbreak in 2001 of cVDPV1. In actuality, the outbreak started in 2000 and continued into 2001. See MMWR 50(39) 855-6, October 5, 2001.*

- This is a correct statement. The abstract and corresponding relevant text have been corrected to align with MMWR reports.

*7.    On page 3, left column, 3rd paragraph, line 11, there is a typo: "a low" should be "as low".*

- Thank you for noting this, it has now been changed in the manuscript.

*8.    There is another typo on page 4, left column, line 10. The authors say "systemtic". Should it be "systematic"?*

- Thank you for noting this, it has now been changed in the manuscript.

*9.    Also in that same paragraph, there are some asterisks. This reviewer did not see what the asterisks referred to.*

- Thank you for noting this, when conducting literature searches in large databases, an asterisk is used to include variations of the same word within the search criteria. For example, “poliovirus*” would include all versions of the word in the search criteria, such as: “polioviruses”. This is standard best practice for systematic reviews, especially when words such as “immunisation” can be spelled differently in the English language. Such a word should be included as “immuni*ation” in searches to include articles that use “immunization” and “immunisation”. 
- For this reason, no changes have been made to the manuscript text regarding search terms.

# Peer review: Reviewer response for version 1

**DOI:** 10.21956/gatesopenres.14511.r30747

**Published:** 2021-07-07

**Authors:** Yvonne Maldonado

**Affiliations:** 1 Department of Pediatrics, Stanford University, Stanford, CA, USA

Thank you for the opportunity to read an interesting paper on the importance of ongoing surveillance for the detection of cVDPVs and the impact of surveillance methodology on the timely detection of mutated vaccine virus. The statistical analyses were sound as described for the research questions and have brought up a number of interesting points for the GPEI to consider, especially in light of the decline in reported cases of AFP in the last year.

1. I would have liked to see more description in the methods regarding the source of the genetic sequences used in this analysis and the measures put into place to assess the validity of these sequences. Would the authors be able to expand further in the main text?
2. In the third paragraph of the methods, the authors note, “Separate datasets were created for serotype 2 and serotypes 1 and 3, and the latter retained a covariate for serotype.” While the authors noted that a covariate for serotype exists in the shared dataset, I was wondering why different datasets were not created for each serotype?
3. Given the impact of vaccination on the emergence of cVDPVs, were the authors able to look at vaccination schemes or vaccination coverage as a covariate for the countries where outbreaks were detected? Or to look at vaccination coverage in these outbreaks in relation to neighboring countries where outbreaks were not detected?
4. Paragraph 6 in the discussion brings up a great point on the value of environmental surveillance as a tool for earlier detection of cVDPVs. I would suggest moving that higher in the discussion.

Is the work clearly and accurately presented and does it cite the current literature?

Yes

If applicable, is the statistical analysis and its interpretation appropriate?

Yes

Are all the source data underlying the results available to ensure full reproducibility?

Partly

Is the study design appropriate and is the work technically sound?

Yes

Are the conclusions drawn adequately supported by the results?

Yes

Are sufficient details of methods and analysis provided to allow replication by others?

Partly

Reviewer Expertise:

Infectious Disease Epidemiology, Polio Epidemiology, Oral Poliovirus Vaccine Transmission, Environmental Surveillance for Oral Poliovirus Vaccine

I confirm that I have read this submission and believe that I have an appropriate level of expertise to confirm that it is of an acceptable scientific standard, however I have significant reservations, as outlined above.

# Response

**Published:** 2022-04-26

**Authors:** Megan Auzenbergs

**Affiliations:** London School of Hygiene & Tropical Medicine, UK

Thank you for your important comments on this analysis. We have taken your questions into consideration and offer the following responses and changes to the manuscript:

*1.    I would have liked to see more description in the methods regarding the source of the genetic sequences used in this analysis and the measures put into place to assess the validity of these sequences. Would the authors be able to expand further in the main text?*

- We have provided further clarification of the sequencing protocol, which is carried out by CDC, in the text (line number 103):
  - “All samples undergo confirmatory testing and genetic sequencing at laboratories that are part of the Global Polio Laboratory Network (GPLN) following a standardised protocol to minimise contamination and maximise sensitivity.”

*2.    In the third paragraph of the methods, the authors note, “Separate datasets were created for serotype 2 and serotypes 1 and 3, and the latter retained a covariate for serotype.” While the authors noted that a covariate for serotype exists in the shared dataset, I was wondering why different datasets were not created for each serotype?*

- Thank you for the clarifying question. The reason was statistically motivated. The sample size for types 1 and 3 were too small to ascertain meaningful summary statistics. Further the case: infection ratio is similar for serotypes 1 and 3, whereas serotype 2 has a larger case to infection ratio.
- Both of these reasons have now been added to the methods section of the manuscript, lines 153-154:
  - “Separate datasets were created for serotype 2 and serotypes 1 and 3, to account for the small sample size of types 1 and 3 outbreaks and because of the similar case to infection ratio for serotypes 1 and 3. The data set for types 1 and 3 retained a covariate for serotype.”

*3.    Given the impact of vaccination on the emergence of cVDPVs, were the authors able to look at vaccination schemes or vaccination coverage as a covariate for the countries where outbreaks were detected? Or to look at vaccination coverage in these outbreaks in relation to neighboring countries where outbreaks were not detected?*

- Routine immunisation coverage was assessed as a potential covariate as we recognised the importance of vaccination schemes. However, the covariate for RI coverage was dropped as inclusion did not result in a model of best fit, as determined by Akaike information criterion (AIC).
- A proposed mechanism by which RI may affect the time from seeding to detection of a VDPV is that in areas of lower RI coverage, there may be an increase in virus circulation, subsequent higher levels of infection and cases, and therefore decreased time to detection.  It is possible that RI coverage has more of an impact at a smaller geographical level than at the national level, the level at which this analysis was carried out. In this case, the analysis at the national level may not detect a relationship for RI coverage and time to detection that may exist at a smaller geographical level. We recognise this as a limitation and potential source of a type 1 error. 
- This limitation has now been included in the discussion section about limitations, lines: 349-352.

*4.    Paragraph 6 in the discussion brings up a great point on the value of environmental surveillance as a tool for earlier detection of cVDPVs. I would suggest moving that higher in the discussion.*

- Thank you for this suggestion. This paragraph has now been moved up higher so it is one of the first points in the discussion, line 282 onwards.
